# Factors that predict outcome of intensive care treatment in very elderly patients: a review

**DOI:** 10.1186/cc3536

**Published:** 2005-05-17

**Authors:** Sophia E de Rooij, Ameen Abu-Hanna, Marcel Levi, Evert de Jonge

**Affiliations:** 1 Head, Department of Geriatrics, Academic Medical Center, University of Amsterdam, Amsterdam; 2 Adjunct Head, Department of Medical Informatics, Academic Medical Center, University of Amsterdam, Amsterdam; 3 Professor and Head, Department of Internal Medicine, Cardiology and Pulmonary Disease, Academic Medical Center, University of Amsterdam, Amsterdam; 4 Adjunct Head Department of Intensive Care, Academic Medical Center, University of Amsterdam, Amsterdam

## Abstract

**Introduction:**

Advanced age is thought to be associated with increased mortality in critically ill patients. This report reviews available data on factors that determine outcome, on the value of prognostic models, and on preferences regarding life-sustaining treatments in (very) elderly intensive care unit (ICU) patients.

**Methods:**

We searched the Medline database (January 1966 to January 2005) for English language articles. Selected articles were cross-checked for other relevant publications.

**Results:**

Mortality rates are higher in elderly ICU patients than in younger patients. However, it is not age *per se *but associated factors, such as severity of illness and premorbid functional status, that appear to be responsible for the poorer prognosis. Patients' preferences regarding life-sustaining treatments are importantly influenced by the likelihood of a beneficial outcome. Commonly used prognostic models have not been calibrated for use in the very elderly. Furthermore, they do not address long-term survival and functional outcome.

**Conclusion:**

We advocate the development of new prognostic models, validated in elderly ICU patients, that predict not only survival but also functional and cognitive status after discharge. Such a model may support informed decision making with respect to patients' preferences.

## Introduction

Projections by the US Census Bureau [1] suggest that the population aged 85 years and older is likely to grow from about 4 million in 2000 to 19 million by 2050. This 'greying' of the population has also been identified in European countries and in Japan. Ageing of the population increases the proportion of people with chronic conditions, with corresponding expectations of eventual decline in function. Advanced age is associated with increased mortality in intensive care unit (ICU) patients [2]. Furthermore, the life expectancy of all elderly patients, remains limited, even after successful ICU treatment. In the UK life expectancy at age 80 years increased from 5.8 years in 1981 to 7.2 years in 2002 for males, and from 7.5 to 8.7 years for females [3]. Thus, the costs per year of life gained, both economical and emotional, are relatively high for elderly patients. Indeed, life-sustaining treatment is more often withdrawn or withheld in older patients. However, few data are available to help identify patients who will benefit from ICU treatment from those who will not.

In this review we focus on the most important factors that may influence outcomes in very elderly critically ill patients, on models that predict short-term and long-term outcome, and on the available data on patients' preferences regarding life-sustaining treatment and how these preferences are influenced by the likelihood of a beneficial outcome.

## Materials and methods

A Medline search (January 1966 to January 2005) was performed using the terms 'frail elderly', 'geriatric', 'very elderly' and 'octogenarians'; and 'critical illness', 'critical care', 'intensive care' and 'intensive care units'; in combination with the terms 'prognosis', 'predictor', or 'outcome'.

Based on title and abstract, we selected English language articles containing clinical data on the outcomes of ICU treatment in very elderly patients. The reference lists of all reports were cross-checked for other potentially relevant articles. In the reports identified in this search, we examined factors that influence outcome in elderly patients such as age, diagnosis, comorbidity, functional status (including cognitive functioning) before hospital admittance, delirium, malnutrition, dehydration, acute renal failure, length of stay, and complications such as nosocomial infections and pressure ulcers. It was envisaged that the studies would be too heterogeneous to combine in a formal meta-analysis, and therefore a narrative synthesis, mainly focusing on prospective studies or very large retrospective studies, was undertaken.

In accordance with published criteria [4], we consider patients aged 80 years and older to be 'very elderly'. However, as several published studies used different criteria for defining a patient as elderly, we also consider data based on studies in other patient groups (e.g. those older than 70 years). Where data specific to elderly patients are not available, we briefly review best knowledge based on studies in patients of all ages.

## Results and discussion

### Factors influencing outcome in elderly patients

#### Age

When discussing the influence of age on ICU outcome, it is important to appreciate that all published studies, either prospective or retrospective, were performed in selected populations of elderly patients after admission to an ICU. Because intensive treatments, including intensive care, are often withheld in elderly patients [4,5], patients with severe comorbidity may be under-represented in these studies. This could result in an over-optimistic view on the effects of age on ICU outcome in the selected patient groups. On the other hand, high mortality rates in the studies may partly be accounted for by decisions to withhold life-sustaining treatments because of advanced age.

For this overview, we consider those patients aged 80 years or older to be 'very elderly' patients, in accordance with the definitions proposed by the SUPPORT (Study to Understand Prognosis and Preferences for Outcomes and Risks of Treatment) investigators [4].

We found 12 prospective cohort studies or retrospective studies based on large databases that addressed the influence of age on outcome in ICU patients (Table 1) [6-17]. In 1995 Cohen and Lambrinos [8] presented the results of a study of the impact age has on outcome of mechanical ventilation in a 41,848-patient, state-wide database. They found that in-hospital mortality in patients receiving mechanical ventilation aged 85 years or older was 70%, as compared with 32% in patients aged 29 years or younger. Only 14% of patients aged 85 years or older went home without home health care, as compared with 47% in patients aged 29 years or younger. Another large retrospective cohort study [9], conducted in data from consecutive ICU admissions to 38 ICUs, showed increased risk for hospital death with more advanced age. Relative to patients younger than 35 years, the adjusted odds of death in patients aged 80–84 years and ≥90 years were 3.9 and 4.7, respectively. These findings were adjusted for severity of illness, Acute Physiology Score, admission source, diagnosis and comorbidity. These conclusions are in accordance with the findings of the SUPPORT study [4]. In that study the risk of death was shown to increase by 1.0% for year of age in patients aged 18–70 years, and by 2.0% for patients aged 70 years or older.

**Figure 1:**
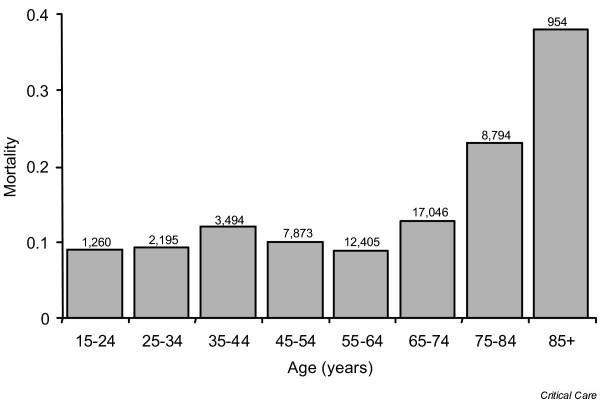
In-hospital mortality by age group in the Dutch National Intensive Care Evaluation database (*n *= 54021) [18]. Numbers indicate patients per age group.

Figure 1 shows the effect of age on in-hospital mortality in 54,021 patients admitted to various ICUs participating in the Dutch National Intensive Care Evaluation (NICE) registry [18]. The in-hospital mortality rate in patients aged 85 years or older was fourfold higher than in patients younger than 65 years.

Although advanced age clearly increases the risk for not surviving an ICU stay, this does not mean that all critically ill elderly patients have a poor prognosis. Studies in specific subgroups of elderly patients have shown that mortality may be as low as 4.3% or 22.1% for patients older than 85 years admitted to a surgical ICU [19,20], 15–25% in neurosurgical ICU patients, and 39–48% for medical ICU patients [21].

Despite potential bias in all studies, many suggest that older patients are more likely to die or experience adverse outcomes of their ICU treatment. However, several studies, using multivariate analysis, showed that age was not an independent predictor of mortality [6,16,21-23]. It appears that it is not advanced age *per se *but other factors associated with advanced age that determine prognosis in elderly patients.

#### Diagnosis

The conclusion that very elderly ICU patients are at substantially increased risk for dying may not hold true for all subgroups of patients. It was found that the effects of age on prognosis very much depend on other factors such as diagnosis. In patients aged 80–84 years hospital mortality was 85% for those with infection as their reason for admission, as compared with 58% for those with diagnoses of gastrointestinal disorder [8]. In another study [24], whereas in-hospital mortality in elderly patients on mechanical ventilation due to pneumonia was 62%, it was 40% in ventilated trauma patients. Outcome after brain injury in geriatric trauma patients is notoriously poor, with mortality and functional disability rates twice those in younger patients [25]. In a general population (all ages), it was shown that 13.6% of the predictive power of the Acute Physiology and Chronic Health Evaluation (APACHE) III model was due to admitting diagnosis [26]. Our data from the Dutch NICE database [18] show that, between 1997 and 2002, in-hospital mortality in ICU patients aged 80 years or older was 16.5% in those who had undergone cardiac surgery but 46% in other patients.

We can conclude that the reason for admission to an ICU has a major influence on prognosis.

#### Comorbidity

Comorbidity, defined as the total burden of illness unrelated to a patient's principal diagnosis, contributes to clinical outcomes (e.g. mortality, surgical results, complication rates, functional status and length of stay) as well as to economic outcomes (e.g. resource utilization, discharge destination and intensity of treatments) [27-29]. Most information on the influence of comorbidity on outcome after ICU admission comes from studies in patients of all ages. In 1987, Charlson and coworkers [29] developed a weighted index of comorbidity that takes into account the number as well as the seriousness of comorbid diseases. This index was shown to predict the 1-year mortality of hospitalized medical patients.

Some studies investigated the relationship between comorbidity and mortality in critically ill patients of all ages. Among the severity of illness models that predict mortality in critically ill patients, comorbidity is included in APACHE II and III [30,31] but not in the Simplified Acute Physiology Score (SAPS) II [32] or Mortality Probability Model (MPM) II [33]. It was shown that the APACHE II model was a very good predictor of mortality in critically ill patients, but that the chronic health points components of APACHE II did not have discriminating ability [34]. Furthermore, it was also shown that the Charlson index had some predictive value in critically ill patients but with an area under the receiver operating characteristic (ROC) curve of only 0.67, indicating limited discriminating ability. In a retrospective cohort study conducted in more than 17,000 ICU patients [35], comorbidity was found to account for only 8.4% of the predictive ability of APACHE II, as compared with 67.7% for laboratory values and 17.7% for diagnosis [35].

Comorbidity is commonly present in elderly patients. However, we could not find any study of the possible influence of comorbidity on outcome conducted specifically in (very) elderly critically ill patients.

#### Functional status before hospital admittance

Functional status, including physical, cognitive and social functioning, has been shown to be an important predictor of the hospital outcomes of older patients. Not surprisingly, impaired functioning in daily life is more likely to be prevalent in older patients and was found to form an independent predictor of mortality [36-38].

Functional status is generally not assessed by physiologically based models such as SAPS II and APACHE II and III. In ICU patients of all ages, an association between functional status and mortality was found by some investigators [39] but not by others [22,40]. Few clinical studies described the value of premorbid functional status in predicting ICU outcomes in the very elderly.

In 1991, Mayer-Oakes and coworkers [41] found in older ICU patients that those who died were significantly more likely to be totally dependent on help for activities of daily living than were those who survived. It was recently reported that long-term survival after admission to a medical ICU is dependent on functional status before admission [5]. In a more recent study [16], the prognosis of elderly patients hospitalized in a medical ICU depended not only on APACHE II scores but also on the loss of functional independence and on the presence of moderate to severe cognitive impairment before ICU admission. Mortality was 30% in patients who had an Activities of Daily Living score of 1–6 (dependent), as compared with 7.8% in patients with a score of 0 (independent). Likewise, mortality was 55.9% in patients with severe cognitive impairment versus 8.2% in those without cognitive impairment. Also, in older patients with severe pneumonia requiring mechanical ventilation, the Activities of Daily Living score before admission was shown to be an important predictor of discharge outcome [42].

Another recent study [22] showed that, in a population of very old patients, mortality after ICU discharge occurred predominantly during the first 3 months.

Although various instruments for measurement of impaired functioning were employed in the reviewed studies, both age and prior limitation of activity were associated with risk for dying during the ICU stay.

In a recent prospective cohort study conducted in 817 adult patients receiving prolonged mechanical ventilation, long-term ICU outcome, defined as mortality after 1 year of follow up, was also found to be associated with advanced age and poor functional status before hospitalization [22,43].

#### Other factors related to intensive care outcome in very elderly patients

Risk adjustment indices, which are mainly based on demographic data, and the existing prognostic models may underestimate the effects on prognosis of complicating conditions that are frequently present in older patients and that are under-reported in administrative databases. Examples of these are malnutrition and delirium.

Low body mass index has been shown to be an independent predictor of in-hospital mortality [36,44,45]. Malnutrition was common in older hospitalized patients with medical illness, and was also associated with delayed functional recovery and higher rates of nursing home use. These adverse outcomes were not accounted for by greater severity of acute illness, comorbidity, or functional dependence in malnourished patients on hospital admission [36]. This relation between nutrition, in some studies expressed as a low body mass index, and mortality was also confirmed in ICU patients aged 65 years and older [16]. Delirium, an often overlooked complication in older ICU patients, is an independent predictor of reintubation, prolonged hospital stay and mortality [46-48].

Other factors that may have an effect on prognosis are complications, such as adverse drug events [49], nosocomial infections [50] and pressure ulcers [16]. However, no studies were found concerning the impacts of these complications on outcome specifically in very elderly critically ill patients treated in ICUs.

### Patient preferences

Patients do not necessarily prefer life-extending treatment over care focused on relieving pain and discomfort. The willingness to receive life-sustaining treatment depends on the burden of treatment, the outcome and the likelihood of the outcome. In a population of patients with limited life expectancy and aged 60 years or older, 74% stated that they would not choose treatment if the burden of treatment were high and the anticipated outcome survival with severe functional impairment [51]. Under the same conditions, 88% of patients opted not to undergo treatment if cognitive impairment was the expected outcome. The number of participants who stated that they would choose treatment declined as the likelihood of an adverse outcome increased. In another study conducted in patients aged 65 years and older [52], patients' willingness to receive cardiopulmonary resuscitation if they suffered a cardiac arrest decreased from 41% to 22% after learning the probability of survival (10–17%). Only 6% of patients aged 86 years or older opted for cardiopulmonary resuscitation under these conditions. Substantial differences in the willingness to receive life-sustaining treatment exist that may depend on ethnicity, religion, the role of family and other variables [53].

Unfortunately, physicians are often unaware of the treatment preferences of their patients. In a study conducted in 4556 patients [4], physicians did not know the preference of their patient in 25% of cases. Furthermore, their assessments of patients preferences were correct for 45% and incorrect for the remaining 30% of patients. Physicians were more likely to believe incorrectly that patients did not want life-extending care when patients were older (79% of the time for patients older than 80 years, as compared with 36% for patients younger than 50 years).

### Prognostic models in intensive care

Patients and their representatives base their decisions regarding what treatments they wish to undergo to a large extent on the likelihood of a favourable outcome. This underscores the importance of reliable information on what outcome can be expected. In order to help physicians to estimate the likelihood of survival of their patients, several severity-of-illness based mortality prediction models were developed for use in multidiagnostic patient groups. They were developed using logistic regression and incorporate information about physiological derangement, admitting diagnosis, age and sometimes comorbid disease. In the general ICU population, these prognostic models, such as SAPS II [32], MPM II [33] and APACHE II and III [30,31], predict the probability of survival of critically ill patients reasonably well.

The information derived from these models can be used to evaluate ICU performance and to improve medical decision making, and perhaps it can also provide patients and their relatives with better information about the ICU stay and its possible outcomes. Unfortunately, when using prognostic models for individual decision making, the risk cannot be ruled out that these models will become self-fulfilling prophecies. If treatment is withdrawn in patients with a high risk for dying, then all high-risk patients indeed will die.

A potential limitation of these models is the fact that they are exclusively based on data obtained during the first 24 hours after ICU admission and that they do not take into account complications that may develop during treatment. It has been shown that the accuracy of prognostic models based on data from the first 24 hours after ICU admission is maintained at an acceptable level only in patients who stay in the ICU for a short period of time [54]. After this period has elapsed discriminative power decreases, probably resulting from excess risk for death associated with acquired infections or other iatrogenic complications during the ICU stay. Different models have been developed that use scores calculated on a daily basis in a general ICU patient population, showing good discriminating value [55,56]. Other potential limitations of prognostic models include the influence of organizational factors on patient outcomes [57,58], between country differences in performance of models [59] and mistakes in data collection [60].

The commonly used prognostic models have not been calibrated for use in the elderly. In a prospective cohort study conducted in patients on mechanical ventilation for pneumonia [61], the predictive values for mortality of the APACHE II, SAPS II and MPM II models were found to be significantly lower for patients aged 75 years or older as compared with younger patients.

Using the technique of recursive partitioning, El Solh and coworkers [42] developed a classification tree to predict hospital mortality in elderly ICU patients with pneumonia. This model exhibited good accuracy, with an area under the ROC of 0.93 versus 0.71 for the APACHE II model. However, that study is limited by the limited number of studied patients (*n *= 104) and the lack of a different population in which to validate the model.

Another model specifically developed to predict mortality and functional outcome in very elderly ICU patients used demographic and physiologic data as well as attributes of ICU treatment and ICU illnesses, such as the use of mechanical ventilation and the development of sepsis [21]. Although the model was developed in a relatively small number of patients (*n *= 243), it exhibited good discriminating performance for short-term outcome (predicting death and discharge to home or to a nursing facility).

## Conclusion

The ICU population is ageing, and it may be concluded that very elderly patients admitted to ICUs represent a distinct and important subgroup of patients. In general, very elderly patients have poorer outcomes than do younger patients, but prognosis is more dependent on severity of illness and functional status before admission than on high age itself. A number of prognostic models have been developed that predict survival in critically ill patients, but these models are not calibrated for use in very old patients. Furthermore, they do not take into account some known risk factors, such as comorbid conditions, and functional and cognitive status before ICU admission. Finally, they do not give a prognosis regarding (long-term) functional status after hospital discharge. We suggest that a model should be developed for predicting outcome of ICU treatment in very old patients, taking into account all discussed prognostic factors. Such a model could more precisely predict the (long-term) discharge outcome of these patients and support informed decision making, in accordance with the preferences of the patients and their relatives.

## Key messages

• 	ICU mortality is higher in elderly patients.

• 	High age alone is not responsible for the poorer outcome, but premorbid functional status and severity of illness also contribute.

• 	Present prognostic models are not suited for elderly individuals

• 	All (premorbid) prognostic factors should be taken into account in a prognostic model to support informed decision making.

## Abbreviations

APACHE = Acute Physiology and Chronic Health Evaluation; ICU = intensive care unit; MPM = Mortality Probability Model; ROC = receiver operating characteristic; SAPS = Simplified Acute Physiology Score.

## Competing interests

The author(s) declare that they have no competing interests

## Authors' contributions

EdJ acquired and interpreted data, and participated in preparing the manuscript. SEdR interpreted data and participated in preparing the manuscript. AA-H analyzed and interpreted data. ML interpreted data. All authors read and approved the final manuscript.

## Figures and Tables

**Table 1:** Studies concerning intensive care outcome and age

Study	Sample	Study type	Age	Main findings
Chelluri *et al*. (1993) [10]	97 ICU patients	Prospective chart investigation	65–74 years (*n *= 43) and ≥75 years (*n *= 54)	Age itself was not an adequate predictor of long-term survival and quality of life, but severity of illness was
Dardaine *et al*. (1995) [7]	110 ICU patients on mechanical ventilation	Prospective cohort study	> 70 years	ICU mortality was 31% and 6-month mortality was 52%; outcome predictors were shock on admission and previous health status
Cohen and Lambrinos (1995) [8]	14,848 ICU patients on mechanical ventilation	Retrospective cohort study	>18 years	In-hospital mortality, in patients receiving mechanical ventilation aged ≥85 years, was 70% versus 32% in patients aged ≤29 years
Dewar *et al*. (1999) [9]	37,573 patients on prolonged mechanical ventilation	Retrospective database analysis	> 18 years	Inverse relation between age and survival; older survivors were often discharged to residential health care facilities
Ely *et al*. (1999) [12]	300 ICU patients	Prospective cohort study	<75 years versus >75 years	No difference in duration of artificial ventilation
Montuclard *et al*. (2000) [13]	75 ICU patients	Prospective cohort study	> 70 years	ICU mortality was 60% in elderly patients receiving ICU treatment
Ely *et al*. (2002) [14]	902 Patients with acute lung injury or ARDS	Prospective cohort study	<70 years (*n *= 729) and >70 years (*n *= 173)	Patients aged 70 years and older were twice as likely to die than were younger patients, and had greater difficulty achieving liberation from the ventilator
Rosenthal *et al*. (2002) [15]	156,136 Consecutive admissions to medical, surgical, neurological, and mixed medical/surgical ICUs	Retrospective cohort study	18–100 years	The adjusted odds of death increased with each 5-year age increment
Djaiani and Ridley (1997) [17]	474 ICU patients	Prospective cohort study	>70 years	The 1-year survival of patients aged <85 years was 56%, which was significantly better than that of patients aged >85 years (27%)
Bo *et al*. (2003) [16]	659 Medical ICU patients	Prospective cohort study	≥ 65 years	Independent predictors of mortality were functional dependence and cognitive impairment before admission, high APACHE II score and low body mass index
Tang *et al*. (2003) [11]	365 ICU patients on mechanical ventilation	Prospective cohort study	≥ 65 years (*n *= 206) and <65 years (*n *= 159)	Severity of acute illness and chronic co-morbidities, but not age, were predictors of medical ICU and hospital mortality in elderly ventilated patients
Chelluri *et al*. (2004) [43]	817 ICU patients on mechanical ventilation	Prospective cohort study	Mean age 65 years	Long-term mortality rate was associated with old age and poor pre-hospitalization functional status
Esteban *et al*. (2004) [62]	5183 ICU patients on mechanical ventilation	International prospective cohort study	>70 years (*n *= 1612)	Patients older than 70 years had higher in-hospital mortality (55%) but similar duration of mechanical ventilation and length of stay
Boumendil *et al*. (2004) [5]	233 ICU patients aged 80 years and older	Prospective cohort study	>80 years	Long-term survival after ICU stay was mainly related to the underlying condition and preadmission functional status
Vosylius *et al*. (2004) [63]	2067 ICU patients	Prospective observational cohort study	>75 years (*n *= 477)	Mortality in elderly patients was higher than in younger patients; most important risk factors were severity of illness, impaired level of conciousness and infection.

APACHE, Acute Physiology and Chronic Health Evaluation; ARDS, acute resppiratory distress syndrome; ICU, intensive care unit.

## References

[B1] US Census Bureau (2000). Population Projections of the United States by Age, Sex, Race, Hispanic Origin and Nativity: 1999–2100.

[B2] Wood KA, Ely EW (2003). What does it mean to be critically ill and elderly?. Curr Opin Crit Care.

[B3] Gastrell J (2004). Annual update: mortality statistics 2001: general. Health Stat Q.

[B4] Hamel MB, Teno JM, Goldman L, Lynn J, Davis RB, Galanos AN, Desbiens N, Connors AF, Wenger N, Phillips RS (1999). Patient age and decisions to withhold life-sustaining treatments from seriously ill, hospitalized adults. SUPPORT Investigators. Study to Understand Prognoses and Preferences for Outcomes and Risks of Treatment. Ann Intern Med.

[B5] Boumendil A, Maury E, Reinhard I, Luquel L, Offenstadt G, Guidet B (2004). Prognosis of patients aged 80 years and over admitted in medical intensive care unit. Intensive Care Med.

[B6] Chelluri L, Pinsky MR, Grenvik AN (1992). Outcome of intensive care of the "oldest-old"; critically ill patients. Crit Care Med.

[B7] Dardaine V, Constans T, Lasfargues G, Perrotin D, Ginies G (1995). Outcome of elderly patients requiring ventilatory support in intensive care. Aging.

[B8] Cohen IL, Lambrinos J (1995). Investigating the impact of age on outcome of mechanical ventilation using a population of 41,848 patients from a statewide database. Chest.

[B9] Dewar DM, Kurek CJ, Lambrinos J, Cohen IL, Zhong Y (1999). Patterns in costs and outcomes for patients with prolonged mechanical ventilation undergoing tracheostomy: an analysis of discharges under diagnosis-related group 483 in New York State from 1992 to 1996. Crit Care Med.

[B10] Chelluri L, Pinsky MR, Donahoe MP, Grenvik A (1993). Long-term outcome of critically ill elderly patients requiring intensive care. JAMA.

[B11] Tang EY, Hsu LF, Lam KN, Pang WS (2003). Critically ill elderly who require mechanical ventilation: the effects of age on survival outcomes and resource utilisation in the medical intensive care unit of a general hospital. Ann Acad Med Singapore.

[B12] Ely EW, Evans GW, Haponik EF (1999). Mechanical ventilation in a cohort of elderly patients admitted to an intensive care unit. Ann Intern Med.

[B13] Montuclard L, Garrouste-Org, Timsit JF, Misset B, De Jonghe B, Carlet J (2000). Outcome, functional autonomy, and quality of life of elderly patients with a long-term intensive care unit stay. Crit Care Med.

[B14] Ely EW, Wheeler AP, Thompson BT, Ancukiewicz M, Steinberg KP, Bernard GR (2002). Recovery rate and prognosis in older persons who develop acute lung injury and the acute respiratory distress syndrome. Ann Intern Med.

[B15] Rosenthal GE, Kaboli PJ, Barnett MJ, Sirio CA (2002). Age and the risk of in-hospital death: insights from a multihospital study of intensive care patients. J Am Geriatr Soc.

[B16] Bo M, Massaia M, Raspo S, Bosco F, Cena P, Molaschi M, Fabris F (2003). Predictive factors of in-hospital mortality in older patients admitted to a medical intensive care unit. J Am Geriatr Soc.

[B17] Djaiani G, Ridley S (1997). Outcome of intensive care in the elderly. Anaesthesia.

[B18] de Jonge E, Bosman RJ, van der Voort PH, Korsten HH, Scheffer GJ, de Keizer NF (2003). Intensive care medicine in the Netherlands, 1997–2001. I. Patient population and treatment outcome [in Dutch]. Ned Tijdschr Geneeskd.

[B19] Margulies DR, Lekawa ME, Bjerke HS, Hiatt JR, Shabot MM (1993). Surgical intensive care in the nonagenarian. No basis for age discrimination. Arch Surg.

[B20] Van Den NN, Vogelaers D, Afschrift M, Colardyn F (1999). Intensive care for very elderly patients: outcome and risk factors for in-hospital mortality. Age Ageing.

[B21] Nierman DM, Schechter CB, Cannon LM, Meier DE (2001). Outcome prediction model for very elderly critically ill patients. Crit Care Med.

[B22] Somme D, Maillet JM, Gisselbrecht M, Novara A, Ract C, Fagon JY (2003). Critically ill old and the oldest-old patients in intensive care: short- and long-term outcomes. Intensive Care Med.

[B23] Rockwood K, Noseworthy TW, Gibney RT, Konopad E, Shustack A, Stollery D, Johnston R, Grace M (1993). One-year outcome of elderly and young patients admitted to intensive care units. Crit Care Med.

[B24] Meinders AJ, van der Hoeven JG, Meinders AE (1996). The outcome of prolonged mechanical ventilation in elderly patients: are the efforts worthwhile?. Age Ageing.

[B25] Jacobs DG, Plaisier BR, Barie PS, Hammond JS, Holevar MR, Sinclair KE, Scalea TM, Wahl W, EAST Practice Management Guidelines Work Group (2003). Practice management guidelines for geriatric trauma: the EAST Practice Management Guidelines Work Group. J Trauma.

[B26] Knaus WA, Wagner DP, Zimmerman JE, Draper EA (1993). Variations in mortality and length of stay in intensive care units. Ann Intern Med.

[B27] Kaplan MH, Feinstein AR (1974). The importance of classifying initial co-morbidity in evaluatin the outcome of diabetes mellitus. J Chronic Dis.

[B28] Greenfield S, Aronow HU, Elashoff RM, Watanabe D (1988). Flaws in mortality data. The hazards of ignoring comorbid disease. JAMA.

[B29] Charlson ME, Pompei P, Ales KL, MacKenzie CR (1987). A new method of classifying prognostic comorbidity in longitudinal studies: development and validation. J Chronic Dis.

[B30] Knaus WA, Draper EA, Wagner DP, Zimmerman JE (1985). APACHE II: a severity of disease classification system. Crit Care Med.

[B31] Knaus WA, Wagner DP, Draper EA, Zimmerman JE, Bergner M, Bastos PG, Sirio CA, Murphy DJ, Lotring T, Damiano A (1991). The APACHE III prognostic system. Risk prediction of hospital mortality for critically ill hospitalized adults. Chest.

[B32] Le Gall JR, Loirat P, Alperovitch A, Glaser P, Granthil C, Mathieu D, Mercier P, Thomas R, Villers D (1984). A simplified acute physiology score for ICU patients. Crit Care Med.

[B33] Lemeshow S, Teres D, Avrunin JS, Gage RW (1988). Refining intensive care unit outcome prediction by using changing probabilities of mortality. Crit Care Med.

[B34] Poses RM, McClish DK, Smith WR, Bekes C, Scott WE (1996). Prediction of survival of critically ill patients by admission comorbidity. J Clin Epidemiol.

[B35] Johnston JA, Wagner DP, Timmons S, Welsh D, Tsevat J, Render ML (2002). Impact of different measures of comorbid disease on predicted mortality of intensive care unit patients. Med Care.

[B36] Covinsky KE, Justice AC, Rosenthal GE, Palmer RM, Landefeld CS (1997). Measuring prognosis and case mix in hospitalized elders. The importance of functional status. J Gen Intern Med.

[B37] Reuben DB, Rubenstein LV, Hirsch SH, Hays RD (1992). Value of functional status as a predictor of mortality: results of a prospective study. Am J Med.

[B38] Inouye SK, Peduzzi PN, Robison JT, Hughes JS, Horwitz RI, Concato J (1998). Importance of functional measures in predicting mortality among older hospitalized patients. JAMA.

[B39] Goldstein RL, Campion EW, Thibault GE, Mulley AG, Skinner E (1986). Functional outcomes following medical intensive care. Crit Care Med.

[B40] Lemeshow S, Teres D, Pastides H, Avrunin JS, Steingrub JS (1985). A method for predicting survival and mortality of ICU patients using objectively derived weights. Crit Care Med.

[B41] Mayer-Oakes SA, Oye RK, Leake B (1991). Predictors of mortality in older patients following medical intensive care: the importance of functional status. J Am Geriatr Soc.

[B42] El Solh AA, Sikka P, Ramadan F (2001). Outcome of older patients with severe pneumonia predicted by recursive partitioning. J Am Geriatr Soc.

[B43] Chelluri L, Im KA, Belle SH, Schulz R, Rotondi AJ, Donahoe MP, Sirio CA, Mendelsohn AB, Pinsky MR (2004). Long-term mortality and quality of life after prolonged mechanical ventilation. Crit Care Med.

[B44] Landi F, Onder G, Gambassi G, Pedone C, Carbonin P, Bernabei R (2000). Body mass index and mortality among hospitalized patients. Arch Intern Med.

[B45] Galanos AN, Pieper CF, Kussin PS, Winchell MT, Fulkerson WJ, Harrell FE, Teno JM, Layde P, Connors AF, Phillips RS (1997). Relationship of body mass index to subsequent mortality among seriously ill hospitalized patients. SUPPORT Investigators. The Study to Understand Prognoses and Preferences for Outcome and Risks of Treatments. Crit Care Med.

[B46] Ely EW (2003). Optimizing outcomes for older patients treated in the intensive care unit. Intensive Care Med.

[B47] Ely EW, Stephens RK, Jackson JC, Thomason JW, Truman B, Gordon S, Dittus RS, Bernard GR (2004). Current opinions regarding the importance, diagnosis, and management of delirium in the intensive care unit: a survey of 912 healthcare professionals. Crit Care Med.

[B48] Ely EW, Gautam S, Margolin R, Francis J, May L, Speroff T, Truman B, Dittus R, Bernard R, Inouye SK (2001). The impact of delirium in the intensive care unit on hospital length of stay. Intensive Care Med.

[B49] Kane SL, Weber RJ, Dasta JF (2003). The impact of critical care pharmacists on enhancing patient outcomes. Intensive Care Med.

[B50] Bochicchio GV, Joshi M, Knorr KM, Scalea TM (2001). Impact of nosocomial infections in trauma: does age make a difference?. J Trauma.

[B51] Fried TR, Bradley EH, Towle VR, Allore H (2002). Understanding the treatment preferences of seriously ill patients. N Engl J Med.

[B52] Murphy DJ, Burrows D, Santilli S, Kemp AW, Tenner S, Kreling B, Teno J (1994). The influence of the probability of survival on patients' preferences regarding cardiopulmonary resuscitation. N Engl J Med.

[B53] Clarfield AM, Gordon M, Markwell H, Alibhai SM (2003). Ethical issues in end-of-life geriatric care: the approach of three monotheistic religions-Judaism, Catholicism, and Islam. J Am Geriatr Soc.

[B54] Lemeshow S, Klar J, Teres D, Avrunin JS, Gehlbach SH, Rapoport J, Rue M (1994). Mortality probability models for patients in the intensive care unit for 48 or 72 hours: a prospective, multicenter study. Crit Care Med.

[B55] Timsit JF, Fosse JP, Troche G, De Lassence A, Alberti C, Garrouste-Orgeas M, Azoulay E, Chevret S, Moine P, Cohen Y (2001). Accuracy of a composite score using daily SAPS II and LOD scores for predicting hospital mortality in ICU patients hospitalized for more than 72 h. Intensive Care Med.

[B56] Ferreira FL, Bota DP, Bross A, Melot C, Vincent JL (2001). Serial evaluation of the SOFA score to predict outcome in critically ill patients. JAMA.

[B57] Rosenberg AL, Hofer TP, Strachan C, Watts CM, Hayward RA (2003). Accepting critically ill transfer patients: adverse effect on a referral center's outcome and benchmark measures. Ann Intern Med.

[B58] Morales IJ, Peters SG, Afessa B (2003). Hospital mortality rate and length of stay in patients admitted at night to the intensive care unit. Crit Care Med.

[B59] Livingston BM, MacKirdy FN, Howie JC, Jones R, Norrie JD (2000). Assessment of the performance of five intensive care scoring models within a large Scottish database. Crit Care Med.

[B60] Polderman KH, Thijs LG, Girbes AR (1999). Interobserver variability in the use of APACHE II scores. Lancet.

[B61] Sikka P, Jaafar WM, Bozkanat E, El Solh AA (2000). A comparison of severity of illness scoring systems for elderly patients with severe pneumonia. Intensive Care Med.

[B62] Esteban A, Anzueto A, Frutos-Vivar F, Alia I, Ely EW, Brochard L, Stewart TE, Apezteguia C, Tobin MJ, Nightingale P (2004). Outcome of older patients receiving mechanical ventilation. Intensive Care Med.

[B63] Vosylius S, Sipylaite J, Ivaskevicius J (2005). Determinants of outcome in elderly patients admitted to the intensive care unit. Age Ageing.

